# Supplementary material for: Dysregulation of homeostatic cytokine receptors drives prolonged T cell activation following acute SARS-CoV-2 infection in humans
Source: Nat Commun. 2025 Nov 27;16:11693. doi: 10.1038/s41467-025-66753-1 (PMC12748759; doi:10.1038/s41467-025-66753-1)
Supplement: Supplementary file 1 — Supplementary Information [file 41467_2025_66753_MOESM1_ESM.pdf]

Supplementary Information for

Dysregulation of homeostatic cytokine receptors drives prolonged T cell activation following acute SARS-CoV-2 infection in humans

Laura Ceglarek[1,2,3], Patrick Taeschler[2], Alp Inci[1,2], Yves Zurbuchen[2], Sarah Adamo[2], Carlo Cervia-Hasler[2], Miro E. Raeber[1,2,3,4], and Onur Boyman[1,2,3,4,5] *

[1] Center for Human Immunology, University of Zurich, Haldeliweg 4, 8044 Zurich, Switzerland

[2] Department of Immunology, University Hospital Zurich, Schmelzbergstrasse 26, 8091 Zurich, Switzerland

[3] Department of Quantitative Biomedicine, University of Zurich, Winterthurerstrasse 190, 8057 Zurich, Switzerland

[4] Faculty of Medicine, University of Zurich, Pestalozzistrasse 3, 8032 Zurich, Switzerland

[5] Faculty of Science, University of Zurich, Winterthurerstrasse 190, 8057 Zurich, Switzerland

* Corresponding author: Onur Boyman, MD, Center for Human Immunology, University of Zurich, Haldeliweg 4, 8044 Zurich, Switzerland. E-mail: onur.boyman@uzh.ch; phone: +41 44 634 1503.

Supplementary Figures

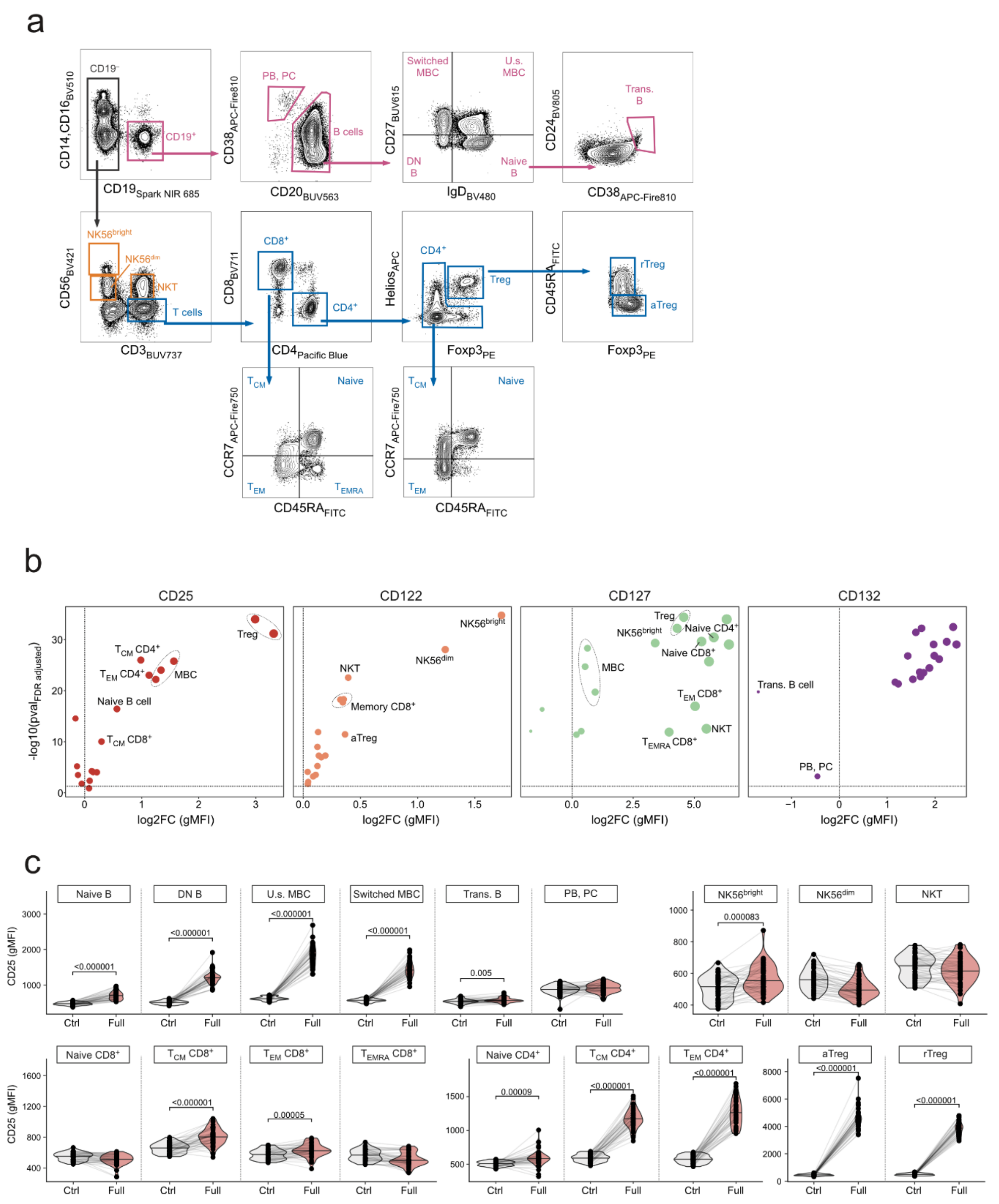

Supplementary Figure 1. Surface abundance of common gamma chain (γc) receptor subunits on different lymphocyte subsets.

a Gating strategy of live lymphocytes. **b** log-2 fold change (log2FC) of CD25, CD122, CD127 and CD132 compared to isotype-matched controls, shown as geometric mean fluorescence intensity (gMFI). Each dot represents a lymphocyte subset. Dashed lines indicate threshold of significantly increased signal compared to controls. Log2FC > 0 and false discovery rate (FDR)-adjusted *p* value of < 0.05 determined by two-tailed Wilcoxon signed-rank test. **c** CD25 expression in indicated human lymphocyte subsets of fully stained samples (Full, $n = 42$) compared to their isotype-matched controls (Ctrl, $n = 42$). aTreg; activated regulatory T; DN B, $CD27^{-}IgD^{-}$ double-negative B; MBC, memory B; NK, natural killer; $NK56^{bright}$, $CD56^{bright}$ natural killer; $NK56^{dim}$, $CD56^{dim}$ natural killer; NKT, natural killer T; PB, plasmablast; PC, plasma cell; rTreg, resting regulatory T; T_{CM}, central-memory T; T_{EM}, effector-memory T; T_{EMRA}, $CD45RA^{+}$ effector-memory T; Trans. B, transitional B; U.s. MBC, unswitched memory B. *P* values were determined by two-tailed, paired t-test and adjusted using the Benjamini-Hochberg method. Source data are provided in the Supplementary Data 1 file.

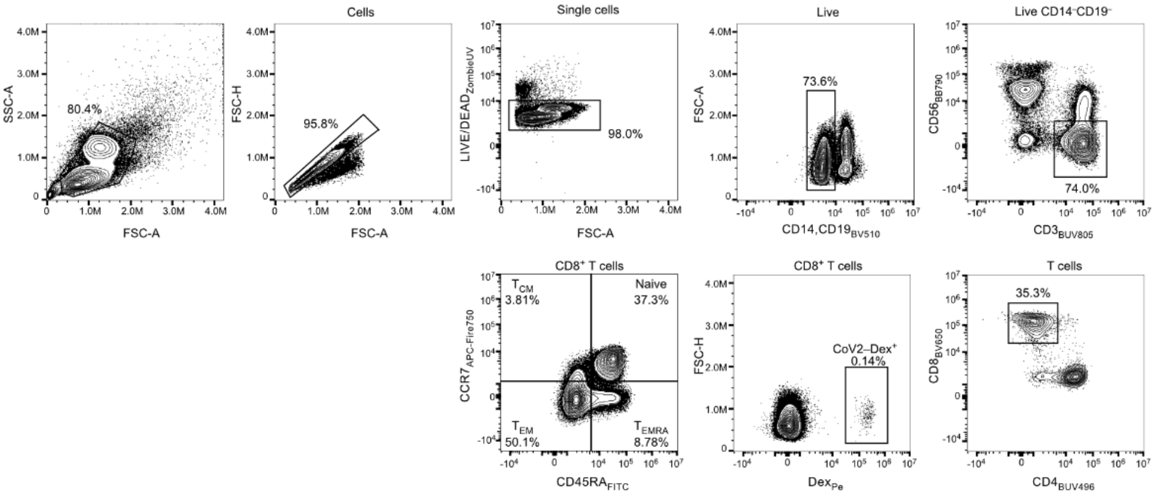

Supplementary Figure 2. Gating strategy for SARS-CoV-2-specific T cells.

Exemplary gating strategy to identify SARS-CoV-2 spike-specific $CD8^+$ T cells by their positive staining for SARS-CoV-2 spike–dextramer (CoV2–Dex) during booster vaccination with the SARS-CoV-2 mRNA vaccine BNT162b2.

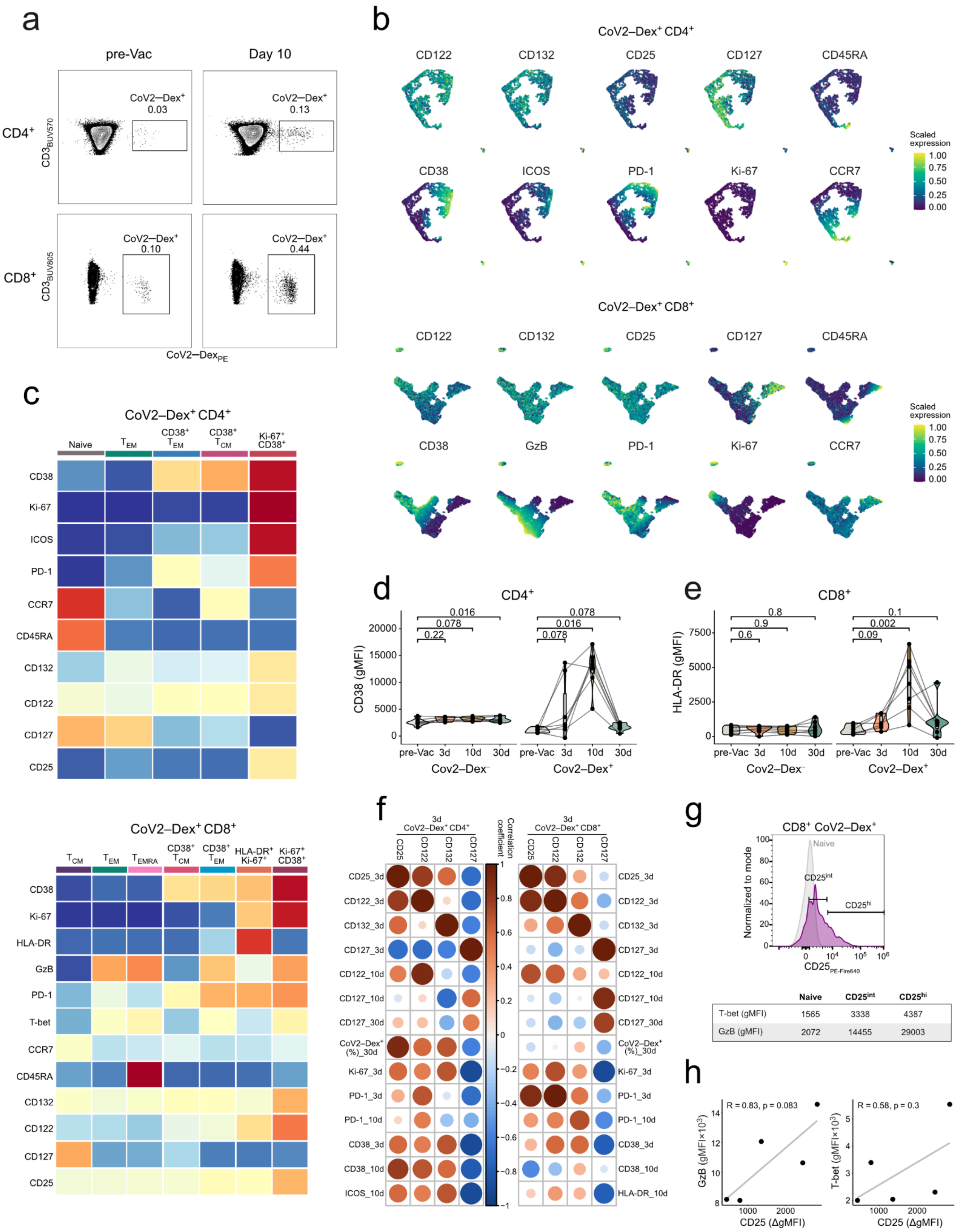
a
pre-Vac
Day 10
CD4+
CD8+
CoV2–Dex+ 0.03
CoV2–Dex+ 0.13
CoV2–Dex+ 0.10
CoV2–Dex+ 0.44
CD3 BUV570
CD3 BUV805
CoV2–Dex PE
b
CoV2–Dex+ CD4+
CD122
CD132
CD25
CD127
CD45RA
CD38
ICOS
PD-1
Ki-67
CCR7
Scaled expression
1.00
0.75
0.50
0.25
0.00
CoV2–Dex+ CD8+
CD122
CD132
CD25
CD127
CD45RA
CD38
GzB
PD-1
Ki-67
CCR7
Scaled expression
c
CoV2–Dex+ CD4+
Naive
TEM
CD38+ TEM
CD38+ TCM
Ki-67+ CD38+
CD38
Ki-67
ICOS
PD-1
CCR7
CD45RA
CD132
CD122
CD127
CD25
CoV2–Dex+ CD8+
TCM
TEM
TEMRA
CD38+ TCM
CD38+ TEM
HLA-DR+ Ki-67+
Ki-67+ CD38+
CD38
Ki-67
HLA-DR
GzB
PD-1
T-bet
CCR7
CD45RA
CD132
CD122
CD127
CD25
d
CD4+
CD38 (gMFI)
0.016
0.078
0.22
0.078
0.016
0.078
pre-Vac
3d
10d
30d
Cov2–Dex−
Cov2–Dex+
e
CD8+
HLA-DR (gMFI)
0.8
0.9
0.6
0.1
0.002
0.09
f
3d CoV2–Dex+ CD4+
3d CoV2–Dex+ CD8+
Correlation coefficient
CD25_3d
CD122_3d
CD132_3d
CD127_3d
CD122_10d
CD127_10d
CD127_30d
CoV2–Dex+ (%)_30d
Ki-67_3d
PD-1_3d
PD-1_10d
CD38_3d
CD38_10d
ICOS_10d
HLA-DR_10d
g
CD8+ CoV2–Dex+
Normalized to mode
Naive
CD25int
CD25hi
CD25 PE-Fire640
Naive
CD25int
CD25hi
T-bet (gMFI)
1565
3338
4387
GzB (gMFI)
2072
14455
29003
h
R = 0.83, p = 0.083
R = 0.58, p = 0.3
GzB (gMFI×10³)
T-bet (gMFI×10³)
CD25 (ΔgMFI)

Supplementary Figure 3. Characterization of SARS-CoV-2-specific T cells following booster vaccination.
a Representative flow cytometry plots of CoV2–Dex^{+} CD4^{+} and CD8^{+} T cells before (pre-Vac) and after (Day 10) vaccination. **b** UMAP of CoV2–Dex^{+} CD4^{+} and CD8^{+} T cells colored by scaled marker expression analyzed by spectral flow cytometry. **c** Heatmap of scaled marker expression in different CoV2–Dex^{+} T cell subsets. **d** CD38 expression on CoV2–Dex^{+} CD4^{+} following booster vaccination (n = 7). Lines connect samples of same individuals. **e** HLA-DR expression on CoV2–Dex^{+} CD8^{+} following booster vaccination (n = 6). Lines connect samples of same individuals. *P* values were determined by two-tailed, paired Wilcoxon signed-rank test and adjusted for multiple comparisons using the Benjamini-Hochberg method. **f** Matrix showing correlation of CD25, CD122, CD132 and CD127 on CoV2–Dex^{+} CD4^{+} (n = 7) and CD8^{+} (n = 6) T cells 3 days post-vaccination with CoV2–Dex^{+} T cell activation at different timepoints after vaccination. The colored bar indicates R values calculated by Spearman's rank correlation coefficient. **g** CD25 expression on pooled CD8^{+} T cells 3 days following booster vaccination (n = 5). SARS-CoV-2 spike-specific CD8^{+} T cells are represented in purple. SARS-CoV-2 spike-specific CD8^{+} T cells were separated into CD25^{hi} (highest 20% of CD25 expression) and CD25^{int} (cells with 20–80% positive CD25 expression) populations, and mean expression of T-box expressed in T cells (T-bet) and granzyme B (GzB) was calculated. **h** Correlation of CD25 induction 3 days following booster vaccination (n = 5), and GzB and T-bet expression on SARS-CoV-2 spike-specific CD8^{+} T cells. Boxplots indicate the median line. The lower and upper hinges correspond to the first and third quartiles. The upper whisker extends from the hinge to the largest value no further than 1.5 IQR. The lower whisker extends from the hinge to the smallest value at most 1.5 × IQR of the hinge. Source data are provided in the Supplementary Data 2 file.

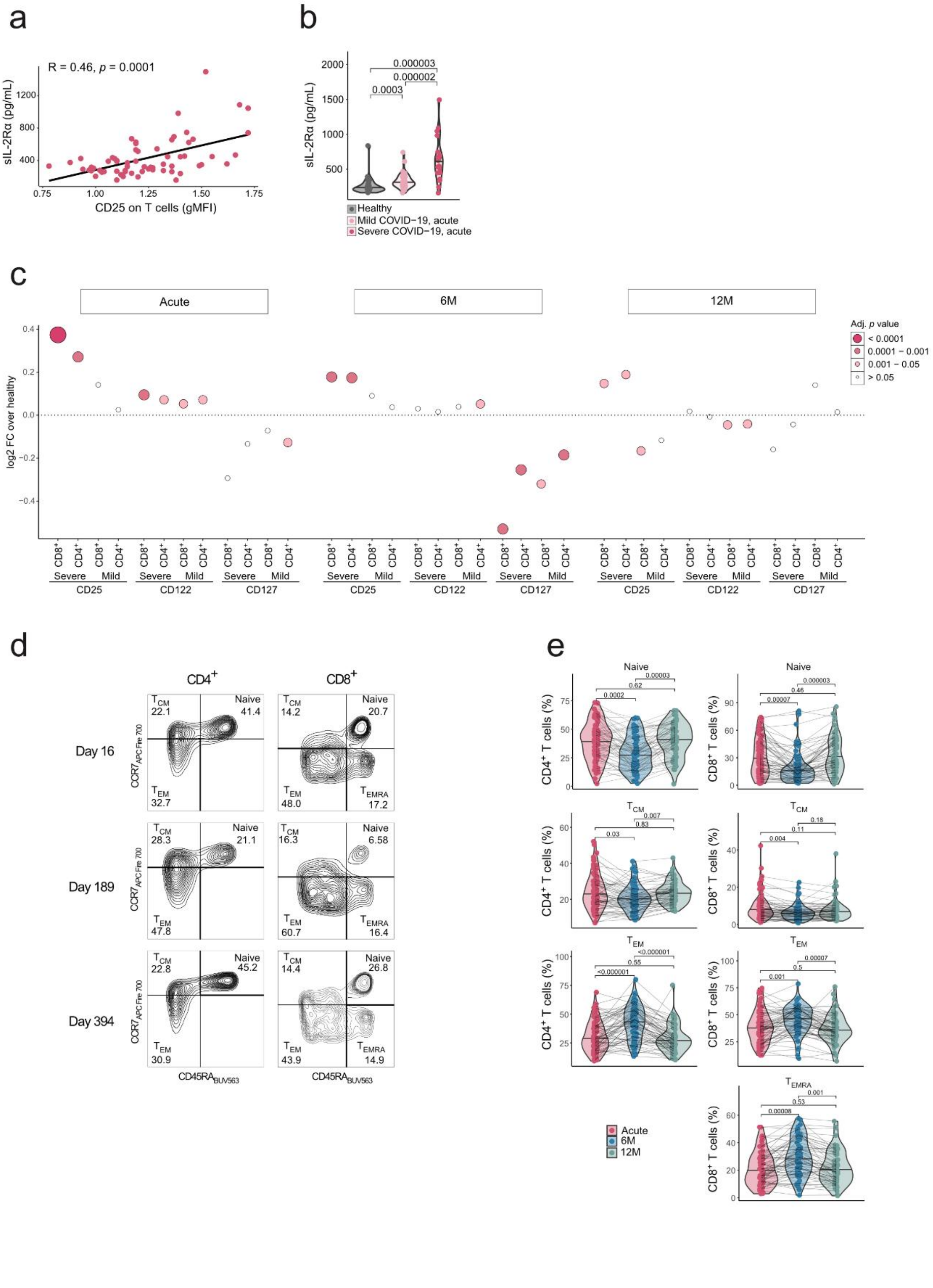

a
R = 0.46, p = 0.0001
sIL-2Rα (pg/mL)
CD25 on T cells (gMFI)
b
sIL-2Rα (pg/mL)
0.0003
0.000002
0.000003
Healthy
Mild COVID−19, acute
Severe COVID−19, acute
c
Acute
6M
12M
log2 FC over healthy
Adj. p value
< 0.0001
0.0001 − 0.001
0.001 − 0.05
> 0.05
Severe
Mild
CD25
CD122
CD127
d
CD4+
CD8+
Day 16
Day 189
Day 394
CCR7 APC Fire 700
CD45RA BUV563
Naive
TCM
TEM
TEMRA
e
Naive
TCM
TEM
TEMRA
CD4+ T cells (%)
CD8+ T cells (%)
Acute
6M
12M

Supplementary Figure 4. $CD4^+$ and $CD8^+$ T cell phenotypes after SARS-CoV-2 infection.

a Correlation of CD25 abundance on T cells with serum concentrations of soluble CD25 (sIL-2Rα) in acute COVID-19 patients (*n* = 64). **b** Serum sIL-2Rα levels in healthy individuals (*n* = 25) as well as in mild and severe COVID-19 patients during acute disease (*n* = 64). *P* values were determined by two-tailed Wilcoxon signed-rank test and adjusted for multiple comparisons using the Benjamini-Hochberg method. **c** Changes in CD25, CD122 and CD127 expression on $CD4^+$ and $CD8^+$ T cells of COVID-19 patients during acute COVID-19 (*n* = 64), and at 6-month follow-up (6M; *n* = 69) and 12-month follow-up (12M; *n* = 66), compared to healthy controls (*n* = 25). Sample groups are depicted in descending absolute differences grouped by disease severity. **d** Representative CCR7 and CD45RA expression in $CD4^+$ and $CD8^+$ T cells obtained from a healthy 38-year-old male subject at indicated timepoints following onset of symptomatic SARS-CoV-2 infection fractions of naive, central memory (T_{CM}), effector-memory (T_{EM}) and $CD45RA^+$ effector-memory (T_{EMRA}) T cell are indicated. **e** Frequencies of indicated $CD4^+$ and $CD8^+$ T cell subsets based on CCR7 and CD45RA expression during acute SARS-CoV-2 infection (*n* = 64), and at 6M (*n* = 69) and 12M follow-up (*n* = 60). *P* values were determined by two-tailed, paired Wilcoxon signed-rank test and adjusted for multiple comparisons using the Benjamini-Hochberg method. Source data are provided in the Supplementary Data 3 file.

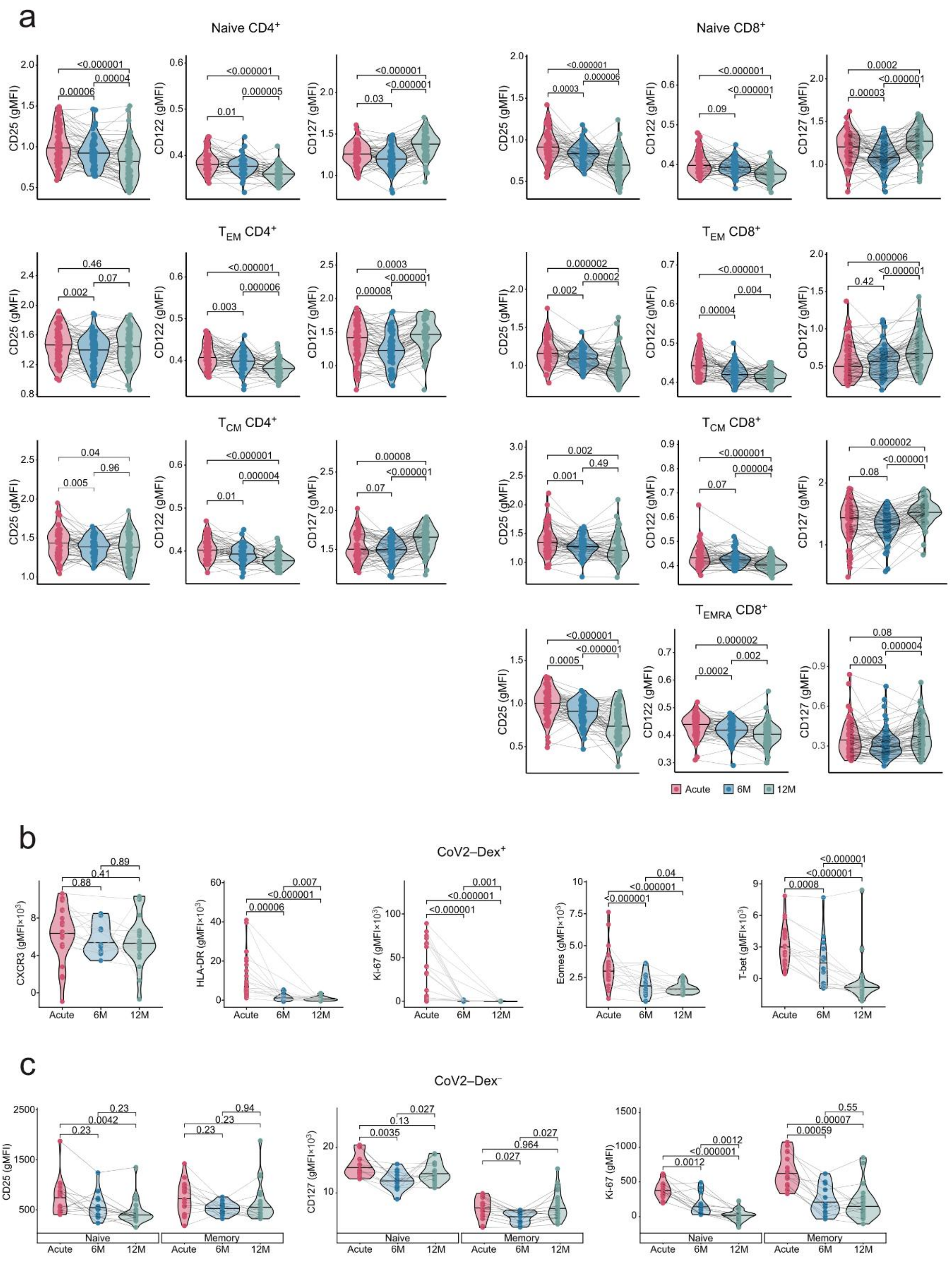
a
Naive CD4+
Naive CD8+
TEM CD4+
TEM CD8+
TCM CD4+
TCM CD8+
TEMRA CD8+
CD25 (gMFI)
CD122 (gMFI)
CD127 (gMFI)
Acute
6M
12M
b
CoV2–Dex+
CXCR3 (gMFI×10³)
HLA-DR (gMFI×10³)
Ki-67 (gMFI×10³)
Eomes (gMFI×10³)
T-bet (gMFI×10³)
c
CoV2–Dex−
CD25 (gMFI)
CD127 (gMFI×10³)
Ki-67 (gMFI)
Naive
Memory

Supplementary Figure 5. IL-2R and IL-7R abundance in $CD4^+$ and $CD8^+$ T cells following COVID-19.

a Surface abundance of CD25, CD122 and CD127 in indicated $CD4^+$ and $CD8^+$ T cell subsets during acute SARS-CoV-2 infection (n = 64), and at 6M (n = 69) and 12M follow-up (n = 60). Gray lines connect data points of same individuals. **b** Effector T cell marker expression on SARS-CoV-2-specific $CD8^+$ T cells during acute COVID-19 (n = 23), and at 6M (n = 15) and 12M follow-up (n = 22). **c** Expression of CD25, CD127 and Ki-67 on naive and memory CoV-2–Dex^- $CD8^+$ T cells during acute COVID-19 (n = 21), and at 6M (n = 21) and 12M follow-up (n = 21). *P* values were determined by two-tailed, paired Wilcoxon signed-rank test and adjusted for multiple comparisons using the Benjamini-Hochberg method. Source data are provided in the Supplementary Data 4 file.

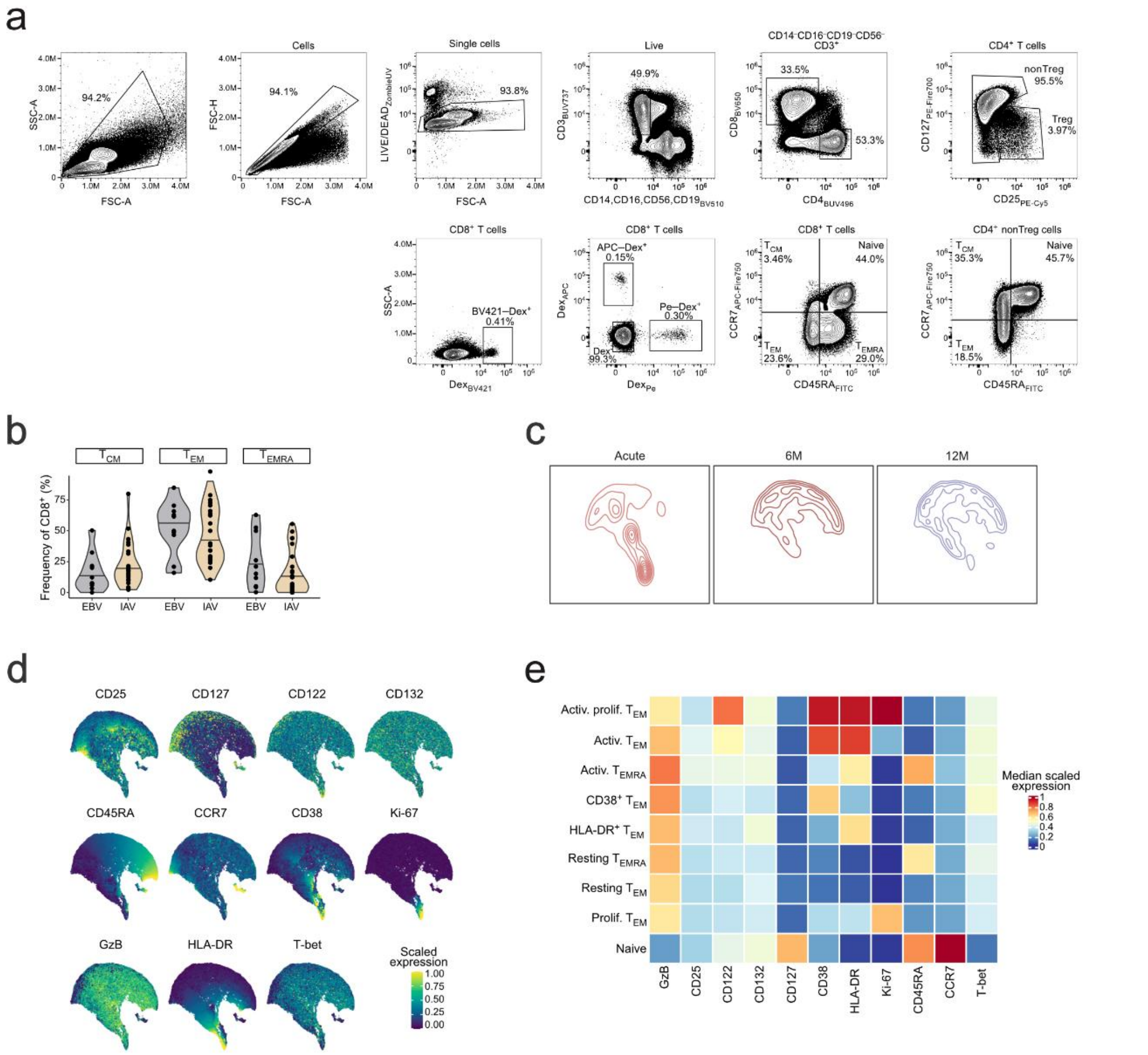

Supplementary Figure 6. Characterization of SARS-CoV-2-, influenza A virus (IAV)-, and Epstein-Barr virus (EBV)-specific CD8$^+$ T cells during and following COVID-19.

a Gating strategy to identify antigen-specific T cell subsets. **b** Frequency of different memory T cell subsets among EBV- (n = 10) and IAV-specific (n = 25) CD8$^+$ T cells at 12M follow-up. **c** Density UMAP of SARS-CoV-2-, IAV-, and EBV-specific CD8$^+$ T cells across indicated timepoints. **d** UMAP projections of SARS-CoV-2-, IAV-, and EBV-specific CD8$^+$ T cells from COVID-19 patients during acute COVID-19, and at 6M and 12M follow-up (n = 26) or healthy individuals (n = 13). Cells are colored by the scaled expression of indicated markers. **e** Heatmap depicting mean scaled marker expression of cells shown in (**d**) split by each identified cell cluster. Source data are provided in the Supplementary Data 5 file.

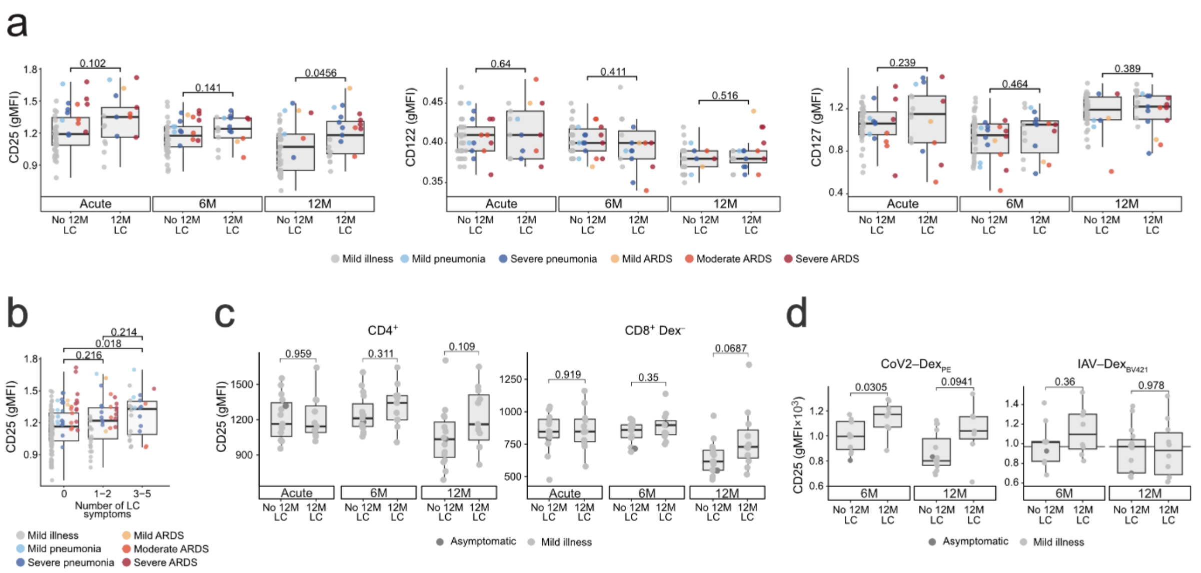

Supplementary Figure 7. IL-2R and IL-7R expression on T cells of patients with active long COVID versus convalescent individuals.

a CD25, CD122 and CD127 expression on total polyclonal T cells during acute COVID-19, 6M and 12M follow-up, in long COVID patients (LC) and convalescent individuals (No LC) with different degrees of initial disease severities, with mild illness in No LC n = 33 and in LC n = 7; mild pneumonia in No LC n = 2 and in LC n = 1; severe pneumonia in No LC n = 5 and in LC n = 3; mild acute respiratory distress syndrome (ARDS) in No LC n = 0 and in LC n = 1; moderate ARDS in No LC n = 3 and in LC n = 2; and severe ARDS in No LC n = 4 and in LC n = 4. **b** CD25 on total polyclonal T cells in convalescent individuals (n = 47) and LC patients exhibiting different numbers of chronic symptoms (1–2 symptoms n = 15; 3–5 symptoms n = 10) across the study period. **c** CD25 on polyclonal $CD4^+$ and CoV2–Dex^- $CD8^+$ T cells in individuals with LC (n = 10) and convalescent individuals (n = 16) that experienced mild or asymptomatic disease. **d** CD25 levels on SARS-CoV-2- and IAV-specific $CD8^+$ T cells at 6M and 12M follow-up in individuals with LC (n = 10) and convalescent individuals (No LC; n = 16) that underwent either mild or asymptomatic disease. Only individuals with more than five CoV2–Dex^+ cells were included in this analysis. P values were determined by two-tailed t-test and adjusted for multiple comparisons using the Benjamini-Hochberg method. Boxplots indicate the median line. The lower and upper hinges correspond to the first and third quartiles. The upper whisker extends from the hinge to the largest value no further than 1.5 IQR. The lower whisker extends from the hinge to the smallest value at most 1.5 × IQR of the hinge. Source data are provided in the Supplementary Data 6 file.

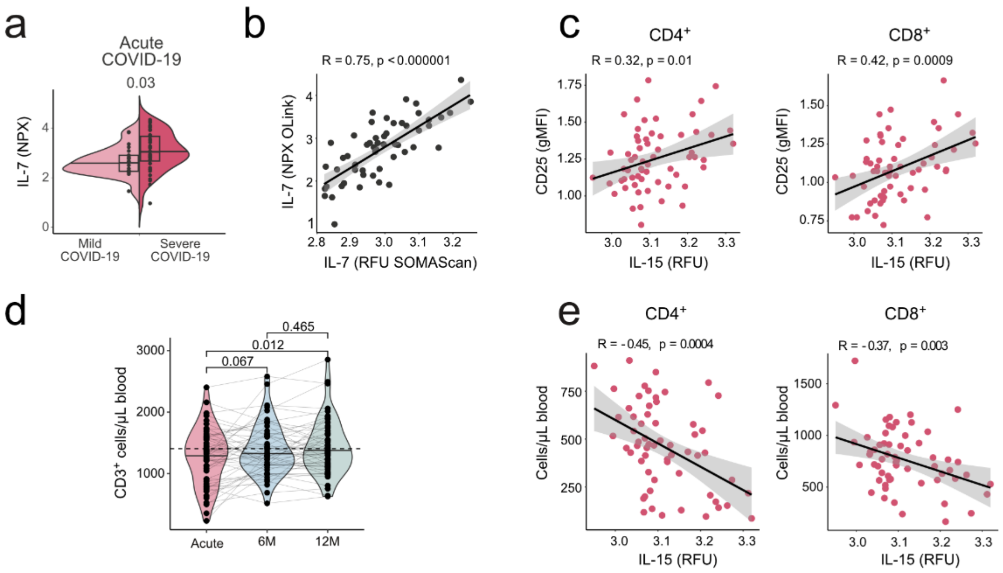

Supplementary Figure 8. Serum concentrations of IL-7 and IL-15 during acute COVID-19.

a Serum IL-7 concentrations in patients exhibiting mild (n = 21) or severe COVID-19 (n = 40), measured using the Olink platform. Boxplots indicate the median line. The lower and upper hinges correspond to the first and third quartiles. The upper whisker extends from the hinge to the largest value no further than 1.5 IQR. The lower whisker extends from the hinge to the smallest value at most 1.5 × IQR of the hinge. **b** Correlation of IL-7 concentrations measured using the Olink platform or an IL-7-specific aptamer of the SomaScan platform. **c** Correlation between surface CD25 abundance and serum IL-15 concentrations of COVID-19 patients during acute infection (n = 64). **d** Blood T cell counts of COVID-19 patients (n = 53) during acute disease, and at 6M and 12M follow-up. **e** Correlation of serum IL-15 concentrations and blood $CD8^+$ or $CD4^+$ T cell counts of COVID-19 patients during acute infection (n = 64). P values were determined by two-tailed, paired Wilcoxon signed-rank test and adjusted for multiple comparisons using the Benjamini-Hochberg method. For **c** and **e**, correlation between variables is visualized with a linear regression, with R indicating the Pearson correlation coefficient with corresponding p value. Serum protein concentrations are indicated in relative fluorescence units (RFU), measured using specific aptamers with the SomaScan platform or in normalized protein expression (NPX) obtained by Olink proteomics. Source data are provided in the Supplementary Data 7 file.

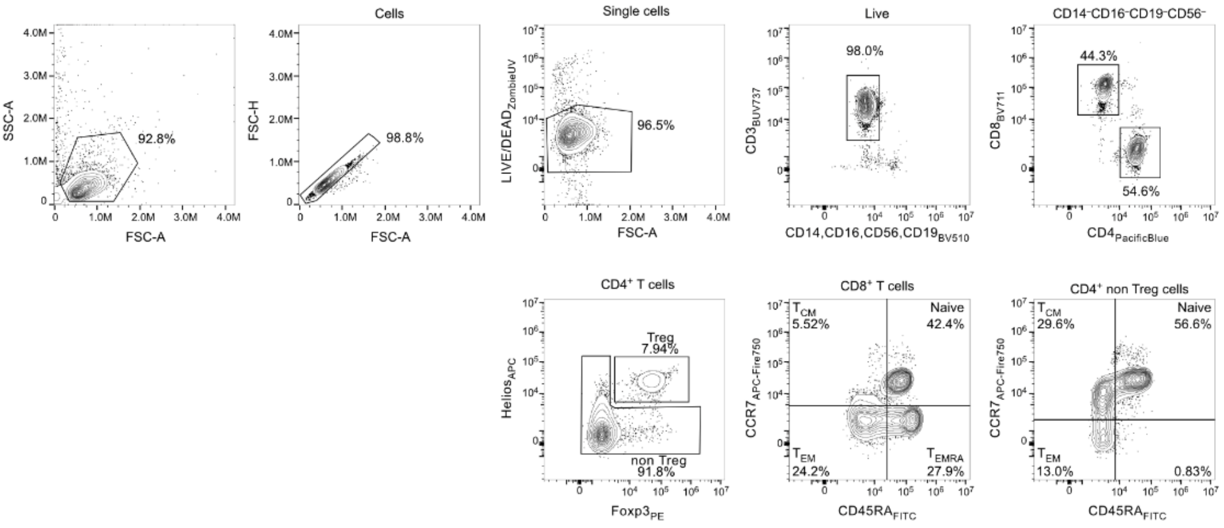

Supplementary Figure 9. Composition of purified T cells before cytokine stimulation *in vitro*.

Exemplary gating strategy and composition of human $CD3^+$ T cells enriched by magnetic-activated cell sorting (MACS) before stimulation with different cytokines *in vitro*.

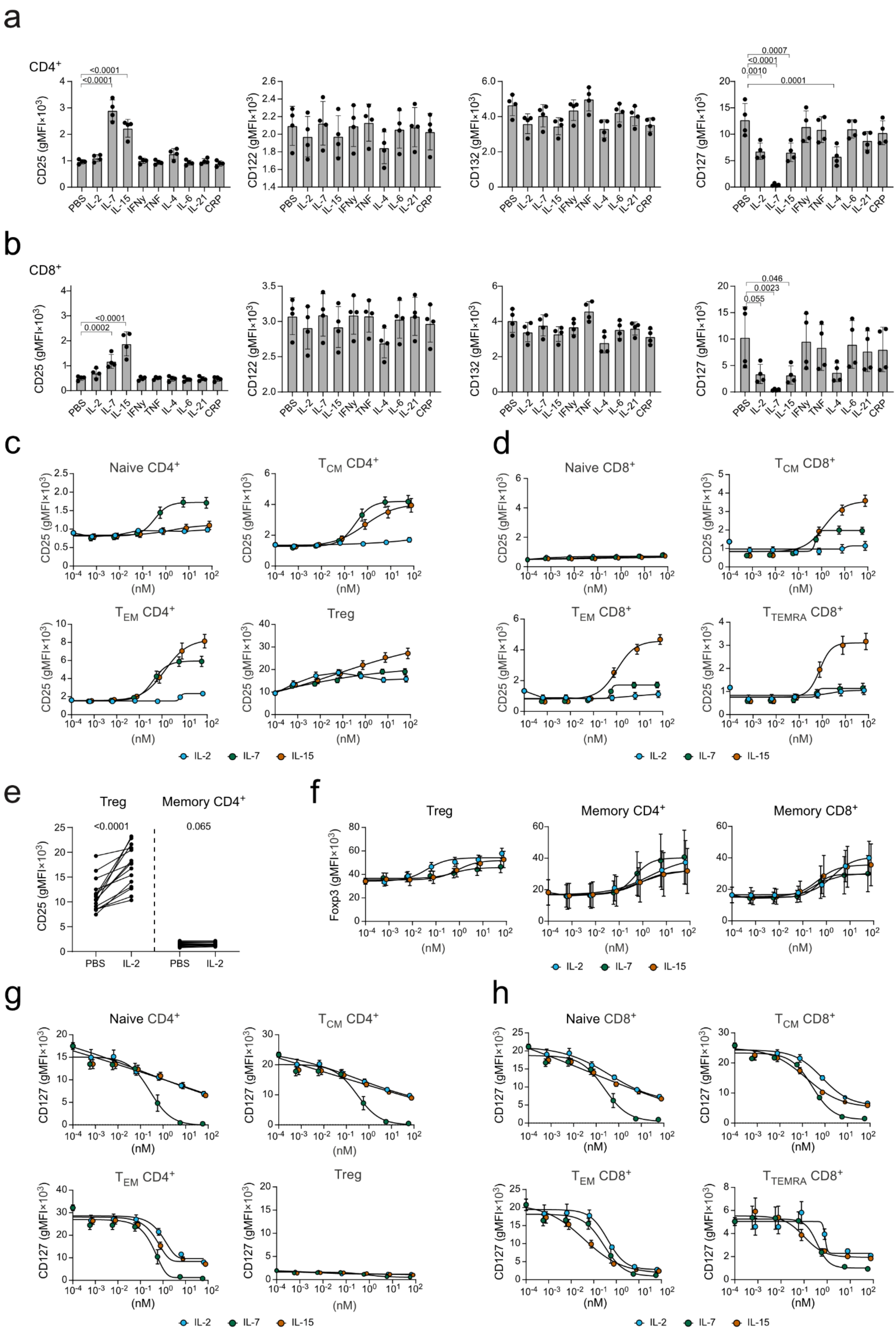
a
CD4+
CD25 (gMFI×10³)
CD122 (gMFI×10³)
CD132 (gMFI×10³)
CD127 (gMFI×10³)
<0.0001
<0.0001
0.0007
<0.0001
0.0010
0.0001
PBS
IL-2
IL-7
IL-15
IFNγ
TNF
IL-4
IL-6
IL-21
CRP
b
CD8+
<0.0001
0.0002
0.046
0.0023
0.055
c
Naive CD4+
TCM CD4+
TEM CD4+
Treg
(nM)
IL-2
IL-7
IL-15
d
Naive CD8+
TCM CD8+
TEM CD8+
TTEMRA CD8+
e
Treg
Memory CD4+
<0.0001
0.065
PBS
IL-2
f
Treg
Memory CD4+
Memory CD8+
Foxp3 (gMFI×10³)
g
Naive CD4+
TCM CD4+
TEM CD4+
Treg
h
Naive CD8+
TCM CD8+
TEM CD8+
TTEMRA CD8+

Supplementary Figure 10. Regulation of IL-2R and IL-7R subunits by γc cytokines *in vitro*.

a, b Modulation of CD25, CD122, CD132 and CD127 expression after stimulation of MACS-purified $CD4^+$. Bars indicate median ± standard deviation (SD). (**a**) and $CD8^+$ (**b**) T cells with indicated cytokines for 48 h *in vitro* ($n = 4$). P values were determined by two-tailed, paired Wilcoxon signed-rank test and adjusted for multiple comparisons using the Benjamini-Hochberg method. **c, d** Modulation of CD25 on different $CD4^+$ (**c**) and $CD8^+$ (**d**) T cell subsets after stimulation with titrated amounts (in nM) of indicated cytokines for 48 h *in vitro* ($n = 15$). **e** Expression of CD25 on Treg cells and memory $CD4^+$ T cells after stimulation with IL-2 (0.65 nM) for 48 h *in vitro* ($n = 15$). **f** Modulation of Foxp3 abundance in Treg cells and memory $CD4^+$ and $CD8^+$ T cells after stimulation with indicated cytokines for 48 h *in vitro* ($n = 15$). **g, h** Modulation of CD127 on different $CD4^+$ (**g**) and $CD8^+$ (**h**) T cell subsets after stimulation with indicated cytokines for 48 h *in vitro* ($n = 15$). Source data are provided in the Supplementary Data 8 file.

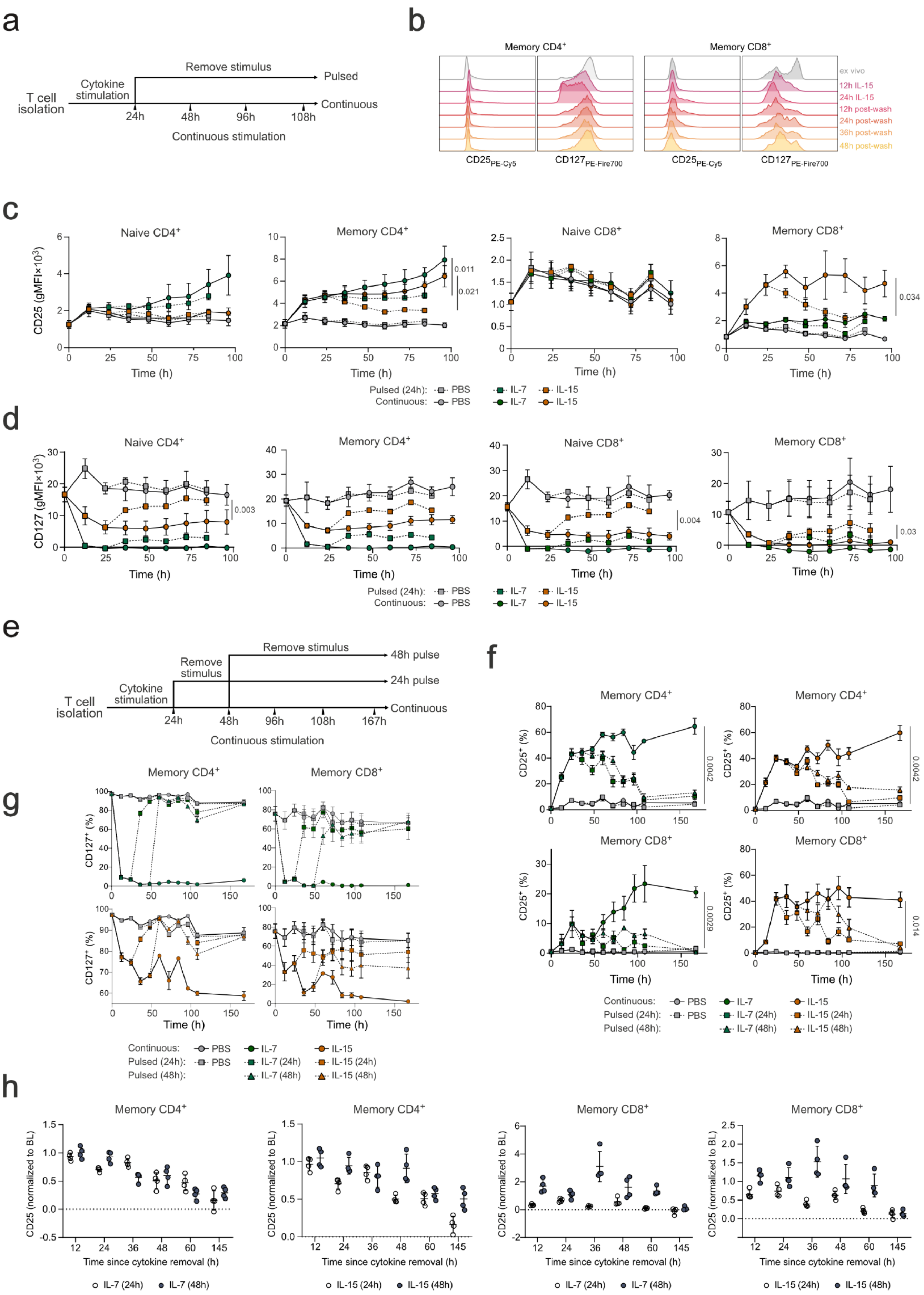
a
T cell isolation
Cytokine stimulation
Remove stimulus
Pulsed
Continuous
24h
48h
96h
108h
Continuous stimulation
b
Memory CD4+
Memory CD8+
ex vivo
12h IL-15
24h IL-15
12h post-wash
24h post-wash
36h post-wash
48h post-wash
CD25 PE-Cy5
CD127 PE-Fire700
c
Naive CD4+
Memory CD4+
Naive CD8+
Memory CD8+
CD25 (gMFI×10³)
Time (h)
0.011
0.021
0.034
Pulsed (24h): PBS IL-7 IL-15
Continuous: PBS IL-7 IL-15
d
CD127 (gMFI×10³)
0.003
0.004
0.03
e
T cell isolation
Cytokine stimulation
Remove stimulus
Remove stimulus
48h pulse
24h pulse
Continuous
24h
48h
96h
108h
167h
Continuous stimulation
f
CD25+ (%)
0.0042
0.0042
0.0029
0.014
Continuous: PBS IL-7 IL-15
Pulsed (24h): PBS IL-7 (24h) IL-15 (24h)
Pulsed (48h): IL-7 (48h) IL-15 (48h)
g
CD127+ (%)
Continuous: PBS IL-7 IL-15
Pulsed (24h): PBS IL-7 (24h) IL-15 (24h)
Pulsed (48h): IL-7 (48h) IL-15 (48h)
h
CD25 (normalized to BL)
Time since cytokine removal (h)
IL-7 (24h) IL-7 (48h)
IL-15 (24h) IL-15 (48h)

Supplementary Figure 11. Changes in IL-2R and IL-7R following continuous stimulation with IL-7 and IL-15 *in vitro*.

a Schematic of *in vitro* stimulation setup. **b** Representative histograms of CD25 and CD127 at indicated timepoints of *in vitro* stimulation. **c**, **d** CD25 (**c**) and CD127 (**d**) on naive and memory T cell subsets after continuous cytokine stimulation (solid lines) or upon stimulation with a 24 h pulse (dashed lines) of indicated cytokines (1 µg/mL, $n = 4$). Stimulation curves were compared by 2-way ANOVA and p values were adjusted for multiple comparisons using Bonferroni correction. **e** Schematic of *in vitro* stimulation setup. **f, g** CD25 (**f**) and CD127 (**g**) on memory T cell subsets after continuous cytokine stimulation (solid lines) or upon stimulation with a 24 h or 48 h pulse (dashed lines) of indicated cytokines (1 µg/mL, $n = 4$). Stimulation curves were compared by 2-way ANOVA and p values were adjusted for multiple comparisons using Bonferroni correction. **h** Comparison of CD25 expression at different times after cytokine removal. CD25 is depicted as fold change over CD25 of unstimulated cells, normalized by CD25 levels at timepoint of cytokine removal after stimulation with indicated cytokines for 24 h or 48 h. Source data are provided in the Supplementary Data 9 file.

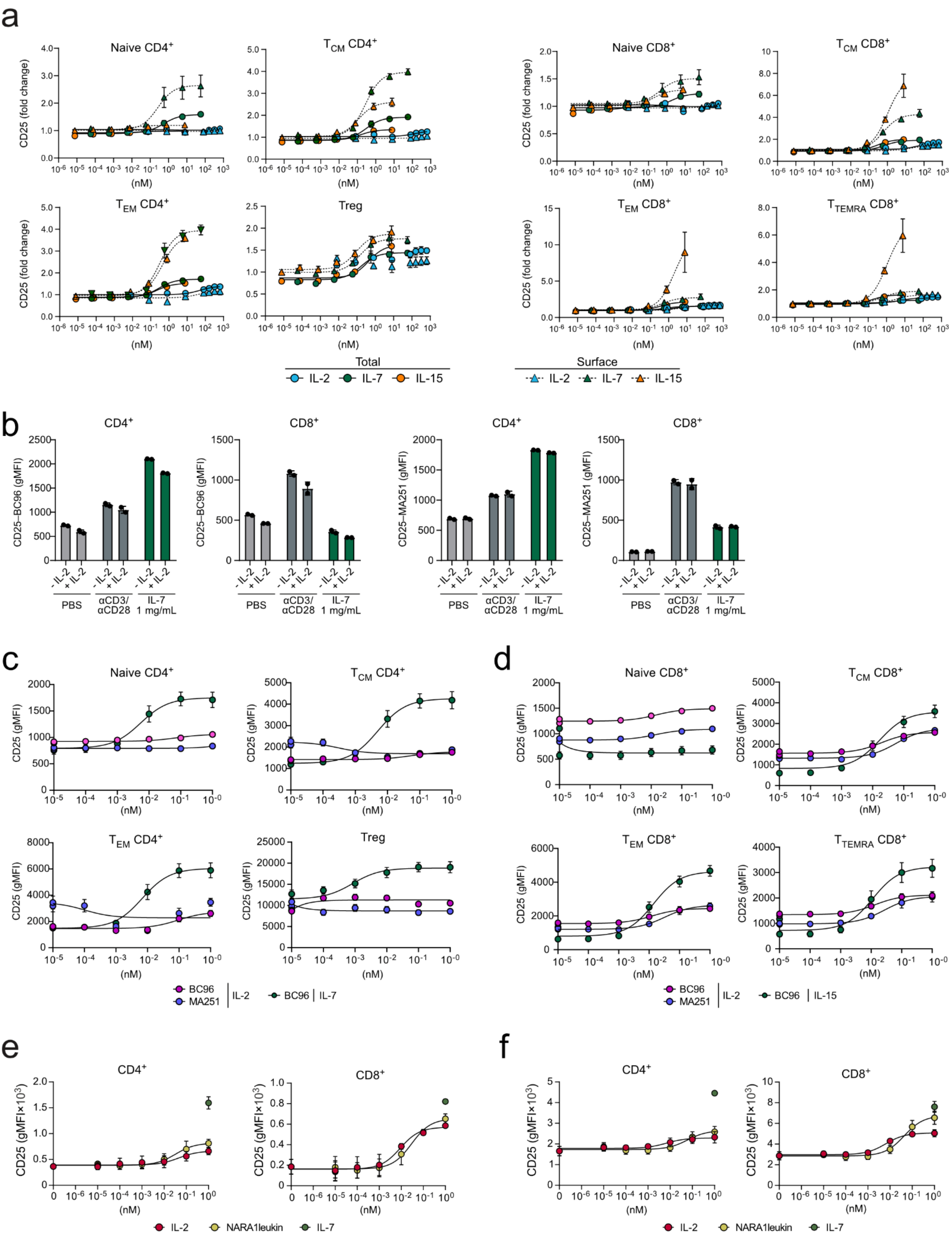
a
Naive CD4+
TCM CD4+
Naive CD8+
TCM CD8+
TEM CD4+
Treg
TEM CD8+
TTEMRA CD8+
CD25 (fold change)
(nM)
Total
IL-2
IL-7
IL-15
Surface
IL-2
IL-7
IL-15
b
CD4+
CD8+
CD25–BC96 (gMFI)
CD25–MA251 (gMFI)
- IL-2
+ IL-2
PBS
αCD3/
αCD28
IL-7
1 mg/mL
c
Naive CD4+
TCM CD4+
TEM CD4+
Treg
CD25 (gMFI)
(nM)
BC96
MA251
IL-2
BC96
IL-7
d
Naive CD8+
TCM CD8+
TEM CD8+
TTEMRA CD8+
CD25 (gMFI)
(nM)
BC96
MA251
IL-2
BC96
IL-15
e
CD4+
CD8+
CD25 (gMFI×10^3)
(nM)
IL-2
NARA1leukin
IL-7
f
CD4+
CD8+
CD25 (gMFI×10^3)
(nM)
IL-2
NARA1leukin
IL-7

Supplementary Figure 12. Modulation of total and surface CD25 by cytokine stimulation *in vitro*.

a Surface CD25 (solid lines) and total CD25 (dashed lines) in memory $CD4^+$ or $CD8^+$ T cells after stimulation with indicated cytokines for 48 h *in vitro* (n = 3). **b** Detection of CD25 by two different αCD25 clones (BC96 and MA251) after stimulation by plate-bound αCD3 and αCD28 antibodies (αCD3/αCD28, 1 µg/mL) or IL-7 for 24 h in the absence (–) or presence (+) of IL-2 (10 µg/mL) in the staining mix. Dots represent technical replicates. Bars indicate median ± standard deviation (SD) **c, d** Detection of CD25 by two different αCD25 clones (BC96, purple; and MA251, blue) on $CD4^+$ (**c**) and $CD8^+$ T cells (**d**) after stimulation with indicated concentrations of IL-2 and IL-7 for 48 h (n = 4). **e, f** Induction of CD25 by stimulation with different concentrations of free IL-2, IL-7, or the IL-2–antibody complex NARA1leukin[1] for 48 h *in vitro* (n = 2) in the absence (**e**) or presence (**f**) of plate-bound αCD3 and αCD28 antibodies (1 µg/mL). Source data are provided in the Supplementary Data 10 file.

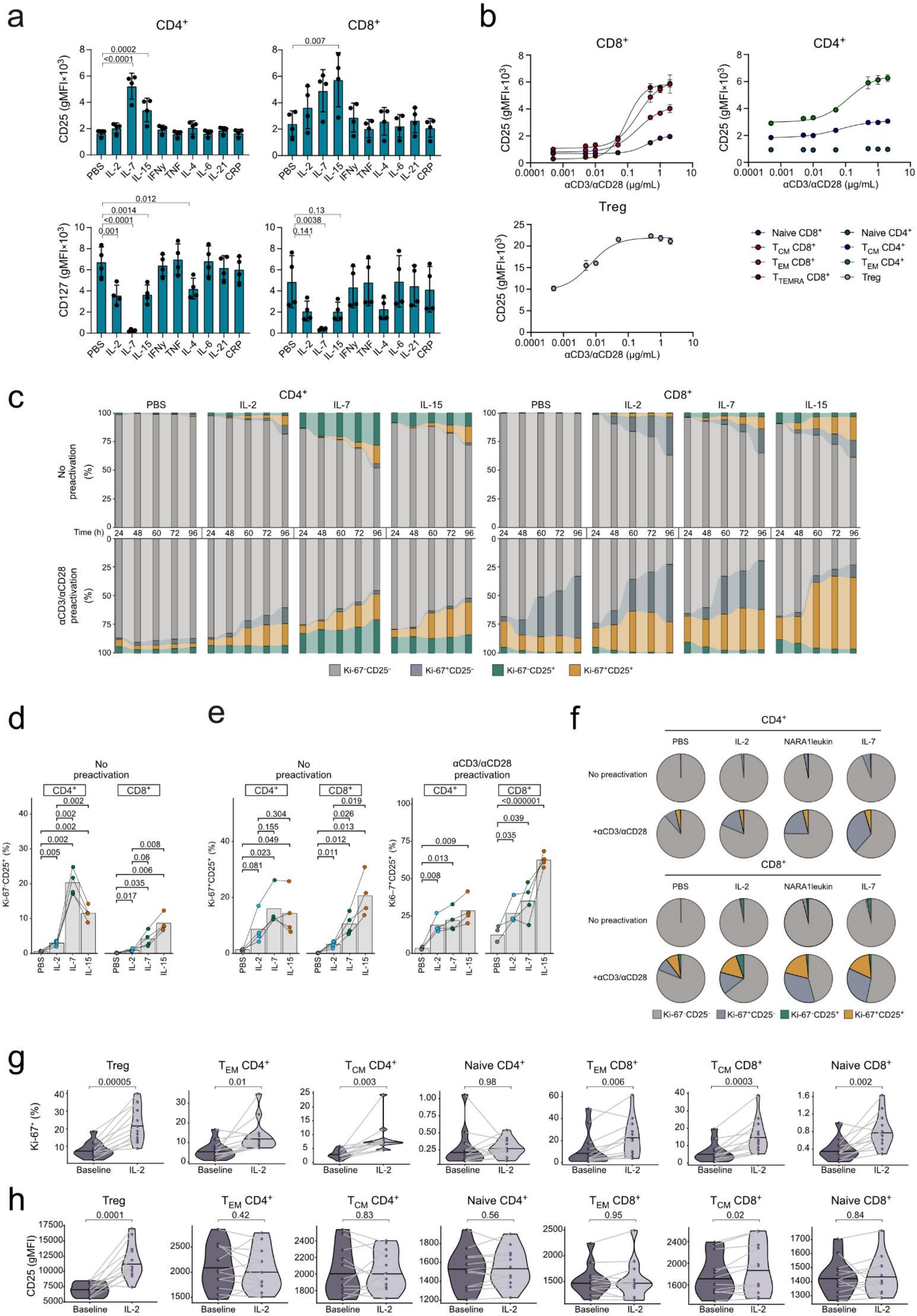

a
CD4+
CD8+
CD25 (gMFI×10³)
CD127 (gMFI×10³)
PBS IL-2 IL-7 IL-15 IFNγ TNF IL-4 IL-6 IL-21 CRP
0.0002
<0.0001
0.007
0.012
0.0014
<0.0001
0.001
0.13
0.0038
0.141
b
CD8+
CD4+
Treg
αCD3/αCD28 (μg/mL)
Naive CD8+
TCM CD8+
TEM CD8+
TTEMRA CD8+
Naive CD4+
TCM CD4+
TEM CD4+
Treg
c
CD4+
CD8+
PBS
IL-2
IL-7
IL-15
No preactivation (%)
αCD3/αCD28 preactivation (%)
Time (h)
Ki-67⁻CD25⁻
Ki-67⁺CD25⁻
Ki-67⁻CD25⁺
Ki-67⁺CD25⁺
d
No preactivation
Ki-67⁻CD25⁺ (%)
e
No preactivation
αCD3/αCD28 preactivation
Ki-67⁺CD25⁺ (%)
Ki6-7⁺CD25⁺ (%)
f
CD4+
CD8+
PBS
IL-2
NARA1leukin
IL-7
No preactivation
+αCD3/αCD28
g
Treg
TEM CD4+
TCM CD4+
Naive CD4+
TEM CD8+
TCM CD8+
Naive CD8+
Ki-67⁺ (%)
0.00005
0.01
0.003
0.98
0.006
0.0003
0.002
Baseline
IL-2
h
CD25 (gMFI)
0.0001
0.42
0.83
0.56
0.95
0.02
0.84

Supplementary Figure 13. Characterization of proliferating T cell subsets upon cytokine stimulation *in vitro* and *in vivo*.

a Modulation of CD25 and CD127 after stimulation of MACS-enriched $CD4^+$ and $CD8^+$ T cells with indicated cytokines and concomitant stimulation with of plate-bound αCD3 and αCD28 crosslinking antibodies for 48 h *in vitro* (n = 4). P values were determined by two-tailed, paired Wilcoxon signed-rank test and adjusted for multiple comparisons using the Benjamini-Hochberg method. Bars indicate median ± standard deviation (SD). **b** Induction of CD25 by stimulation of different T cell subsets with indicated titrated concentrations of plate-bound αCD3 and αCD28 antibodies for 24 h *in vitro*. Error bars indicate standard deviation of two technical replicates. **c** Stacked bar plots of relative frequencies of $CD25^+$ and proliferating (Ki-67^+) T cell subsets during continuous stimulation with indicated cytokines after preactivation (n = 4). **d**, **e** Frequencies of Ki-67^- $CD25^+$ $CD4^+$ and $CD8^+$ T cells after 48 h (**d**) and after 96 h (**e**) of stimulation with indicated cytokines without or with αCD3 and αCD28 preactivation *in vitro* (n = 4). **f** Pie charts depicting relative frequencies of Ki-67^+ and $CD25^+$ T cells after activation with indicated stimuli, including no stimulus, IL-2, IL-7, or the IL-2–antibody complex NARA1leukin, for 48 h *in vitro* (n = 2). **g, h** Frequency of Ki-67^+ (**g**) and CD25 gMFI (**h**) within indicated T cell subsets from SLE patients (n = 12) before (baseline visit) and after receiving five doses of 1.5 million international units of recombinant human IL-2 on five consecutive days, as described by Raeber et al.[2] P values were determined by two-tailed, paired t-test and adjusted for multiple comparisons using the Benjamini-Hochberg method. Source data are provided in the Supplementary Data 11 file.

Supplementary Tables

Supplementary Table 1. Characteristics of healthy and SARS-CoV-2-infected individuals. For continuous variables, medians and interquartile ranges (in parentheses) are shown, and *p* values were obtained by Mann-Whitney U test, compared to healthy individuals. For categorical variables, numbers of individuals and percentages of corresponding subgroup (in parentheses) are specified, with *p* values calculated by two-sided Fisher's exact test, in comparison to healthy individuals. The weighted comorbidity index was calculated according to Charlson et al.[3] and aggregated for indicated cohort subgroups. n.s., not significant; *, $p < 0.05$; **, $p < 0.01$; ***, $p < 0.001$; ****, $p < 0.0001$. OD, optical density; S1, SARS-CoV-2 spike S1 protein. Source data are provided in the Supplementary Data 12 file.

		**Healthy controls**	**Acute SARS-CoV-2**	**6-month follow-up**	**12-month follow-up**
Patient characteristics	n	42	64	69	66
	Mild COVID-19		43 (67.2)	45 (65.2)	48 (72.7)
	Severe COVID-19		21 (32.8)	24 (34.8)	18 (27.3)
	Days after symptom onset		11 (7–17)	199 (185–214)	374 (362–387)
	Age (years)	34 (27–52)	39 (31–59)<br>n.s.	41 (32–61)<br>n.s.	40 (31–58)<br>n.s.
	Sex (female)	22 (52.0)	33 (51.6)<br>n.s.	36 (52.2)<br>n.s.	35 (53.0)<br>n.s.
	SARS-CoV-2-vaccinated	0	0	6 (8.7)	46 (69.7)
Laboratory parameters	Lymphocytes (per µl)	1844 (1524–2283)	1730 (1354–2099)<br>n.s.	1831 (1622–2305)<br>n.s.	1878 (1623–2440)<br>n.s.
	CRP (mg/l)	0.7 (0.4–1.6)	3 (0.7–45.0)<br>**	0.8 (0.6–1.9)<br>n.s.	1.5 (0.7–3.8)<br>*
	IL-6 (ng/l)	0.5 (0–1.6)	2.9 (0.7–17.4)<br>**	1.3 (0.7–2.9)<br>n.s.	1.2 (0.3–2.7)<br>n.s.
	S1-specific IgG (OD ratio)	0.2 (0.15–0.28)	1.0 (0.3–5.1)<br>****	4.0 (1.8–7.5)<br>****	10 (7.2–10)<br>****
	S1-specific IgA (OD ratio)	0.3 (0.3–0.5)	3.0 (0.8–7.4)<br>****	2.8 (1.6–6.0)<br>****	10 (4.6–10)<br>****
Comorbidities	Hypertension	3 (12.0)	12 (18.8)<br>n.s.	14 (20.2)<br>n.s.	13 (19.7)<br>n.s.
	Diabetes mellitus	1 (4.0)	6 (9.4)<br>n.s.	6 (8.7)<br>n.s.	6 (9.1)<br>n.s.
	Heart disease	1 (4.0)	6 (9.4)<br>n.s.	9 (13.0)<br>n.s.	6 (9.1)<br>n.s.
	Lung disease	4 (16.0)	11 (17.2)<br>n.s.	13 (18.8)<br>n.s.	11 (16.7)<br>n.s.
	Kidney disease	0 (0)	4 (6.3)<br>n.s.	4 (5.8)<br>n.s.	5 (7.6)<br>n.s.
	Cancer	1 (4.0)	1 (1.6)<br>n.s.	2 (2.9)<br>n.s.	2 (3.0)<br>n.s.
	Immuno-suppression	0 (0)	3 (4.7)<br>n.s.	3 (4.3)<br>n.s.	2 (3.0)<br>n.s.
	Aggregated Comorbidity Index	10	44<br>n.s.	50<br>n.s.	50<br>n.s.

Supplementary Table 2. Fluorochrome-labeled monoclonal antibodies used for spectral flow cytometry. Panel numbers indicate combination of monoclonal antibodies used in different experiments, including *ex vivo* quantification of cytokine receptor subunits in healthy individuals and COVID-19 patients (panel 1), *in vitro* T cell stimulation (panel 2), HLA-A*02:01 SARS-CoV-2 multimer staining (panel 3), dextramer staining for bystander-activated T cell subsets (panel 4), and SARS-CoV-2 dextramer staining of *ex vivo* immunization responses of $CD4^+$ (panel 5) and $CD8^+$ T cells (panel 6).

Antigen	Fluorophore	Clone	Provider	Cat. # no.	Dilution	Panel
Bcl6	AF647	K112-91	BD Pharmingen	561525	1:100	1
CCR4	BV480	1G1	BD Optibuild	746361	1:100	1,3,5
CCR6	BUV661	11A9	BD Optibuild	750696	1:100	1,3,5
CCR7	APC-Fire750	G043H7	Biolegend	353246	1:50	1,2,4
CCR7	AlexaFluor700	150503	BD Horizon	561143	1:100	3
CCR10	BB515	1B5	BD Horizon	564769	1:100	1
CD122	BV605	Mik-beta3	BD Horizon Customs	624290	1:100	1,3–6
CD122	BB515	MIk-β3	BD Horizon	566059	1:100	2
CD126	PE-Cy7	UV4	Biolegend	352810	1:100	1,2
CD127	PE/Fire 700	A019D5	Biolegend	351365	1:100	1–6
CD132	BV786	TUGh4	BD Horizon	743443	1:100	2,4–6
CD14	Spark Blue 550	63D3	Biolegend	367147	1:400	1,2
CD14	BV510	M5E2	Biolegend	301841	1:200	2–5
CD16	BV510	3G8	Biolegend	302048	1:100	2–4
CD154	BV785	24-31	Biolegend	310842	1:100	1
CD19	BB630	HIB19	BD Horizon Customs	624294	1:400	1
CD19	Spark NIR 685	HIB19	Biolegend	302270	1:100	2
CD19	BV510	HIB19	Biolegend	302241	1:200	2–5
CD25	PE-Cy5	BC96	Biolegend	302608	1:100	1,2,4–6
CD25	PE-F640	M-A251	Biolegend	356147	1:100	3
CD3	BV570	UCHT1	Biolegend	300435	1:50	1,2,5
CD3	BUV 737	UCHT1	BD Horizon	612750	1:200	3,4
CD38	APC-Fire810	HIT2	Biolegend	303550	1:200	1,3–6
CD4	BUV496	SK3	BD Horizon	612936	1:200	1,3–5
CD4	Pacific Blue	SK3	Biolegend	344620	1:100	2
CD45RA	BUV563	HI100	BD Horizon	612927	1:200	1,2
CD45RA	FITC	HI100	Biolegend	304106	1:400	2–4
CD56	BB790	NCAM16.2	BD Horizon Customs	624296	1:400	1–3
CD56	BUV563	NCAM16.2	BD Horizon	612928	1:200	5
CD56	BV510	HCD56	Biolegend	318339	1:200	2,4
CD8	BUV661	SK1	BD Horizon	741683	1:200	1,2
CD8	BV711	SK1	Biolegend	344734	1:200	2
CD8	PerCP	SK1	Biolegend	344708	1:100	3

CD8	BUV805	SK1	BD Horizon	612889	1:200	3,5
CD95	BV480	DX2	BD Horizon	746675	1:100	3
CXCR3	BV650	G025H7	Biolegend	353729	1:100	1,5
CXCR5	BV750	J252D4	Biolegend	356942	1:100	1,3
Foxp3	BV421	206D	Biolegend	320124	1:50	1,3,5
Foxp3	PE	206D	Biolegend	320108	1:100	2
GATA3	PE	TWAJ	Invitrogen	12-9966-41	1:100	1
GzB	PE-Cy7	QA16A02	Biolegend	372213	1:200	4
Helios	APC	22F6	Biolegend	137222	1:100	2
HLA-DR	BV510	L243	Biolegend	307645	1:100	1,2
HLA-DR	BUV615	G46-6	BD Horizon	751142	1:200	3,4
ICOS	BUV615	DX29	BD Optibuild	751092	1:200	1,3,5
Ki-67	BUV395	B56	BD Horizon	564071	1:100	1–6
PD1	BUV737	EH12.1	BD Horizon	612791	1:100	1,2,3,5
PD1	BV650	EH12.1	BD Horizon	564104	1:100	2
RORγt	R718	Q21-559	BD Horizon	567097	1:100	1
T-bet	BV711	4B10	Biolegend	644819	1:100	1–3,5

Supplementary Table 3. MHC dextramers used for detection of virus-specific T cells by spectral flow cytometry.

**HLA type**	**Multimer type**	**Peptide**	**Specificity**	**Fluo-rescent tag**	**Provider**	**Cat. #**
A*02:01	Dextramer	YLQPRTFLL	SARS-CoV-2 spike 269–277	PE	Immudex	WB05824 PE
A*01:01	Dextramer	LTDEMIAQY	SARS-CoV-2 nucleocapsid A1 865–873	PE	Immudex	WA05846 PE 50
DRB1*15:01	Tetramer	NLLLQYGSFCT QLNRAL	SARS-CoV-2 spike 751–767	PE	ProImmune	TT4414-2A
DRB1*04:01	Dextramer	NFSQILPDPSKO SKRSFIED	SARS-CoV-2 spike 801–820	PE	In house	–
DRB1*07:01	Dextramer	KGIYQTSNFRV	SARS-CoV-2 spike 310–320	PE	In house	–
HLA-B*07:02	Dextramer	RPPIFIRRL	EBV-EBNA3 379-387	APC	Immudex	WH02166 APC 50
HLA-B*07:02	Dextramer	SPRWYFYYL	SARS-CoV-2 nucleocapsid 105–113	PE	Immudex	WH05842 PE 50
HLA-A*01:01	Dextramer	VSDGGPNLY	Influenza A PB1 591–599	BV421	Immudex	WA03411 BV421 50
HLA-A*01:01	Dextramer	LTDEMIAQY	SARS-Cov-2 spike 865–973	PE	Immudex	WA05846 PE 50
HLA-A*01:01	Dextramer	TTDPSFLGRY	SARS-Cov-2 ORF1 1637–1646	PE	Immudex	WA05972 PE 50
HLA-A*03:01	Dextramer	ILRGSVAHK	Influenza A nucleoprotein 265–273	BV421	Immudex	WC03814 BV421 50
HLA-A*24:02	Dextramer	TYQWIIRNW	Influenza A PB2 549–557	BV421	Immudex	WF06305 BV421 50

Supplementary Material References

1. Sahin, D. *et al.* An IL-2-grafted antibody immunotherapy with potent efficacy against metastatic cancer. *Nat. Commun.* **11**, 6440 (2020).
2. Raeber, M. E. *et al.* Interleukin-2 immunotherapy reveals human regulatory T cell subsets with distinct functional and tissue-homing characteristics. *Immunity* **57**, 2232-2250.e10 (2024).
3. Charlson, M. E., Pompei, P., Ales, K. L. & MacKenzie, C. R. A new method of classifying prognostic comorbidity in longitudinal studies: Development and validation. *J. Chronic Dis.* **40**, 373–383 (1987).
