# Supplementary material for: Dysregulation of homeostatic cytokine receptors drives prolonged T cell activation following acute SARS-CoV-2 infection in humans
Source: Nat Commun. 2025 Nov 27;16:11693. doi: 10.1038/s41467-025-66753-1 (PMC12748759; doi:10.1038/s41467-025-66753-1)
Supplement: Supplementary file 2 — Description of Additional Supplementary Files [file 41467_2025_66753_MOESM2_ESM.pdf]

Title: Supplementary Data 1

Description: Excel file containing source data for Supplementary Figure 1.

Title: Supplementary Data 2

Description: Excel file containing source data for Supplementary Figure 3.

Title: Supplementary Data 3

Description: Excel file containing source data for Supplementary Figure 4.

Title: Supplementary Data 4

Description: Excel file containing source data for Supplementary Figure 5.

Title: Supplementary Data 5

Description: Excel file containing source data for Supplementary Figure 6.

Title: Supplementary Data 6

Description: Excel file containing source data for Supplementary Figure 7.

Title: Supplementary Data 7

Description: Excel file containing source data for Supplementary Figure 8.

Title: Supplementary Data 8

Description: Excel file containing source data for Supplementary Figure 10.

Title: Supplementary Data 9

Description: Excel file containing source data for Supplementary Figure 11.

Title: Supplementary Data 10

Description: Excel file containing source data for Supplementary Figure 12.

Title: Supplementary Data 11

Description: Excel file containing source data for Supplementary Figure 13.

Title: Supplementary Data 12

Description: Excel file containing source data for Supplementary Table 1.
