# Supplementary material for: Dysregulation of homeostatic cytokine receptors drives prolonged T cell activation following acute SARS-CoV-2 infection in humans
Source: Nat Commun. 2025 Nov 27;16:11693. doi: 10.1038/s41467-025-66753-1 (PMC12748759; doi:10.1038/s41467-025-66753-1)
Supplement: Supplementary file 15 — Reporting Summary [file 41467_2025_66753_MOESM15_ESM.pdf]

nature portfolio

Corresponding author(s): Onur Boyman

Last updated by author(s): 24.10.2025

# Reporting Summary

Nature Portfolio wishes to improve the reproducibility of the work that we publish. This form provides structure for consistency and transparency in reporting. For further information on Nature Portfolio policies, see our Editorial Policies and the Editorial Policy Checklist.

## Statistics

For all statistical analyses, confirm that the following items are present in the figure legend, table legend, main text, or Methods section.

| n/a | Confirmed | |
|---|---|---|
| ☐ | ☒ | The exact sample size (*n*) for each experimental group/condition, given as a discrete number and unit of measurement |
| ☒ | ☐ | A statement on whether measurements were taken from distinct samples or whether the same sample was measured repeatedly |
| ☐ | ☒ | The statistical test(s) used AND whether they are one- or two-sided<br>*Only common tests should be described solely by name; describe more complex techniques in the Methods section.* |
| ☒ | ☐ | A description of all covariates tested |
| ☐ | ☒ | A description of any assumptions or corrections, such as tests of normality and adjustment for multiple comparisons |
| ☐ | ☒ | A full description of the statistical parameters including central tendency (e.g. means) or other basic estimates (e.g. regression coefficient) AND variation (e.g. standard deviation) or associated estimates of uncertainty (e.g. confidence intervals) |
| ☐ | ☒ | For null hypothesis testing, the test statistic (e.g. *F*, *t*, *r*) with confidence intervals, effect sizes, degrees of freedom and *P* value noted<br>*Give P values as exact values whenever suitable.* |
| ☒ | ☐ | For Bayesian analysis, information on the choice of priors and Markov chain Monte Carlo settings |
| ☒ | ☐ | For hierarchical and complex designs, identification of the appropriate level for tests and full reporting of outcomes |
| ☐ | ☒ | Estimates of effect sizes (e.g. Cohen's *d*, Pearson's *r*), indicating how they were calculated |

*Our web collection on statistics for biologists contains articles on many of the points above.*

## Software and code

Policy information about availability of computer code

| | |
|---|---|
| Data collection | For flow cytometry data Cytek Spectroflo was used for data acquisition. Serum proteomics was performed with the SomaScan platform (version 4). |
| Data analysis | FloJo (verison 10.10.0) was used for manual gating of flow cytometry data. R (version 4.4.1) was used for data visualization and statistical testing. Unbiased clustering of flow cytometry data was performed with CATALYST (version 1.28.0) and FlowSOM (version 2.12.0). Batch normalization of flow cytometry data was performed using R (version 4.1.3) and FlowVS (version 1.26.0) as well as CytoNorm (version 0.0.7). Code is available at https://github.com/BoymanLab. |

For manuscripts utilizing custom algorithms or software that are central to the research but not yet described in published literature, software must be made available to editors and reviewers. We strongly encourage code deposition in a community repository (e.g. GitHub). See the Nature Portfolio guidelines for submitting code & software for further information.

## Data

Policy information about availability of data

All manuscripts must include a data availability statement. This statement should provide the following information, where applicable:

- Accession codes, unique identifiers, or web links for publicly available datasets
- A description of any restrictions on data availability
- For clinical datasets or third party data, please ensure that the statement adheres to our policy

Full datasets are available from the corresponding author upon reasonable request. The minimal dataset required to reproduce all figures and conclusions of the

manuscript are deposited under the Zenodo accession code doi.org/10.5281/zenodo.17377036.

## Research involving human participants, their data, or biological material

Policy information about studies with human participants or human data. See also policy information about sex, gender (identity/presentation), and sexual orientation and race, ethnicity and racism.

| | |
|---|---|
| Reporting on sex and gender | The SARS-CoV-2 infection cohort consisted of 36 female and 33 male patients. The healthy control cohort for SARS-CoV-2 infection consisted of 13 female and 12 male subjects. The healthy control cohort depicted in Figure 1 consisted of 22 females and 20 males. The vaccination cohort consistet of 4 female and 6 male individuals. Sex was collected from the electronic medical records, gender data was not collected. |
| Reporting on race, ethnicity, or other socially relevant groupings | Biological samples were obtained from the general population of Switzerland, independent of race, ethnicity or socially relevant groupings. |
| Population characteristics | The cohort characteristics are shown in Supplementary Table 1. |
| Recruitment | Patients at four hospitals in the Canton of Zurich, Switzerland, that had a reverse-transcriptase polymerase chain reaction confirmed SARS-CoV-2 infection and were symptomatic, were approached whether they would be interested in participating in the study. Patients had to be over 18 years old and had to be competent at the time of consent. Following written informed consent the COVID-19 patients donated blood and serum samples. Subsequently, patients visited again at month 6 and 12 post-infection and donated blood and serum samples at the respective time points. The patients were included in the study during their acute disease between April 2020 and September 2020 and for the 12 months follow-up between April 2021 and September 2021. As the patients had to be competent when providing the informed consent this might have skewed the disease severity distribution of the cohort excluding individuals who were mechanically ventilated at the time of positive qPCR. |
| Ethics oversight | The study was approved by the Cantonal Ethical Committee of Zurich (BASEC #2016-01440) and all participants signed a written informed consent before inclusion into the study. |

Note that full information on the approval of the study protocol must also be provided in the manuscript.

# Field-specific reporting

Please select the one below that is the best fit for your research. If you are not sure, read the appropriate sections before making your selection.

☒ Life sciences ☐ Behavioural & social sciences ☐ Ecological, evolutionary & environmental sciences

For a reference copy of the document with all sections, see nature.com/documents/nr-reporting-summary-flat.pdf

# Life sciences study design

All studies must disclose on these points even when the disclosure is negative.

| | |
|---|---|
| Sample size | Sample size was based on the availability of samples rather than on a pre-defined sample size calculation. |
| Data exclusions | For assessing geometric mean of fluorescence and frequencies of antigen-specific T cells, only samples were included were more than antigen-specific 5 cells were detected. |
| Replication | Samples from each patient and timepoint were analyzed once due to limited sample availability. In vitro assays were performed with multiple donors in independent experiments where indicated. |
| Randomization | In this observational study randomization was not applicable. |
| Blinding | As the patients were included based on their SARS-CoV-2 infection history, no blinding was performed. |

# Reporting for specific materials, systems and methods

We require information from authors about some types of materials, experimental systems and methods used in many studies. Here, indicate whether each material, system or method listed is relevant to your study. If you are not sure if a list item applies to your research, read the appropriate section before selecting a response.

## Materials & experimental systems

| n/a | Involved in the study |
|---|---|
| [ ] | [x] Antibodies |
| [x] | [ ] Eukaryotic cell lines |
| [x] | [ ] Palaeontology and archaeology |
| [x] | [ ] Animals and other organisms |
| [x] | [ ] Clinical data |
| [x] | [ ] Dual use research of concern |
| [x] | [ ] Plants |

## Methods

| n/a | Involved in the study |
|---|---|
| [x] | [ ] ChIP-seq |
| [ ] | [x] Flow cytometry |
| [x] | [ ] MRI-based neuroimaging |

# Antibodies

Antibodies used: All flow cytometry antibodies used in this study are indicated in the Supplementary Table 2. Fluorochrome-labelled dextramer reagents for the identification of antigen-specific cells are indicated in Supplementary Table 3.

Validation: The antibodies were validated by the respective manufacturer (see below).
Biolegend:
- Flow Cytometry: The producers tests specificity on 1-3 target cell types with either single- or multi-color analysis (including positive and negative cell types). All the antibodies used had a verified human reactivity. Stainings of human PBMCs are shown as flow plots or histograms on the website. Further each new lot is tested to perform with similar intensity to the in-date reference lot. Brightness (MFI) is evaluated from both positive and negative populations. Each lot product is validated by QC testing with a series of titration dilutions.
BD Bioscience (Flow Cytometry):
Tested for flow cytometry application and verified human reactivity. Stainings of human PBMCs are shown as flow plots or histograms on the website for BD Horizon antibodies.

# Plants

Seed stocks: N/A

Novel plant genotypes: N/A

Authentication: N/A

# Flow Cytometry

## Plots

Confirm that:

- [x] The axis labels state the marker and fluorochrome used (e.g. CD4-FITC).
- [x] The axis scales are clearly visible. Include numbers along axes only for bottom left plot of group (a 'group' is an analysis of identical markers).
- [x] All plots are contour plots with outliers or pseudocolor plots.
- [x] A numerical value for number of cells or percentage (with statistics) is provided.

## Methodology

Sample preparation: Blood was collected from patients and subsequently PBMCs were isolated using a Ficoll density gradient centrifugation, before being washed, counted and frozen in fetal bovine serum (FBS) with 10% dimethyl sulfoxide (DMSO) and stored in liquid nitrogen until use. For subsequent analysis the frozen cells were thawed in pre-warmed R10 medium and subsequently processed for flow cytometry, as described in the methods section.

Instrument: Samples were acquired on a Cytek Aurora.

Software: Flow cytometry data was generated using Cytek SpectroFlo (Version 3.0.3). The flow cytometry data were analysed using FlowJo (version 10.10.0).

| | |
|---|---|
| Cell population abundance | Cell population abundance and purity was determined by flow cytometric analysis of the entire cell sample following in vitro culturing where applicable. |
| Gating strategy | Full gating strategies are shown in Supplementary Figures S1, S2, S6 and S9. |

☒ Tick this box to confirm that a figure exemplifying the gating strategy is provided in the Supplementary Information.
